# Comparison of Myosepta Development and Transcriptome Profiling between Blunt Snout Bream with and Tilapia without Intermuscular Bones

**DOI:** 10.3390/biology10121311

**Published:** 2021-12-10

**Authors:** Jia-Jia Zhou, Yong-Jie Chang, Yu-Long Chen, Xu-Dong Wang, Qing Liao, Rui-Hui Shi, Ze-Xia Gao

**Affiliations:** 1 College of Fisheries, Key Lab of Freshwater Animal Breeding, Ministry of Agriculture, Key Lab of Agricultural Animal Genetics, Breeding and Reproduction of Ministry of Education, Huazhong Agricultural University, No. 1 Shizishan Street, Hongshan District, Wuhan 430070, China; J-2020@webmail.hzau.edu.cn (J.-J.Z.); changyj@webmail.hzau.edu.cn (Y.-J.C.); cyl76@webmail.hzau.edu.cn (Y.-L.C.); wangxudong@webmail.hzau.edu.cn (X.-D.W.); liangqwang@webmail.hzau.edu.cn (Q.L.); 2 Guangdong Laboratory for Lingnan Modern Agriculture, Guangzhou 510000, China; 3 Engineering Research Center of Green Development for Conventional Aquatic Biological Industry in the Yangtze River Economic Belt, Ministry of Education, Wuhan 430070, China

**Keywords:** intermuscular bone, development, *Megalobrama amblycephala*, *Oreochromis niloticus*, histological structure, transcriptome, gene expression

## Abstract

**Simple Summary:**

The presence or absence of intermuscular bones (IBs) is directly related to the economic and edible value of fish. The specific regulatory mechanism of IB formation is not completely known yet. Here, we explored the molecular mechanisms that regulate the formation of IBs based on histological analysis, transcriptome profiling, and gene expression quantification using *M. amblycephala* (with IBs) and *O. niloticus* (without IBs) as models. As a result, we identified several bone-related genes and elucidated their regulatory roles in the development of IBs.

**Abstract:**

Intermuscular bones (IBs) are small spicule-like bones located in the myosepta of basal teleosts, which negatively affect the edibleness and economic value of fish. Blunt snout bream (*Megalobrama amblycephala*, with epineural and epipleural IBs) and tilapia (*Oreochromis niloticus*, without epineural and epipleural IBs) are two major aquaculture species and ideal models for studying the formation mechanisms of fish IBs. Here, we compared myosepta development between *M. amblycephala* and *O. niloticus*, based on histological analysis, transcriptome profiling, and expression analysis of bone-related genes. The histological results showed that dye condensation began to appear in the myosepta 20 days post hatching (dph) in *M. amblycephala*, and IBs could be clearly observed 50 dph in the myosepta, based on different staining methods. However, in *O. niloticus*, dye condensation was not observed in the myosepta from 10 to 60 dph. Differentially expressed genes (DEGs) at different developmental stages were screened by comparing the transcriptomes of *M. amblycephala* and *O. niloticus*, and KEGG analysis demonstrated that these DEGs were enriched in many bone-related pathways, such as focal adhesion, calcium, and Wnt signaling pathways. Quantitative PCR was performed to further compare the expression levels of some bone-related genes (*scxa*, *scxb*, *runx2a*, *runx2b*, *bgp*, *sp7*, *col1a2*, *entpd5a*, *entpd5b*, *phex*, *alpl*, and *fgf23*). All the tested genes (except for *alpl*) exhibited higher expression levels in *M. amblycephala* than in *O. niloticus*. A comparison of the dorsal and abdominal muscle tissues between the two species also revealed significant expression differences for most of the tested genes. The results suggest that *scxa*, *scxb*, *runx2a*, *runx2b*, *entpd5a*, *col1a2*, and *bgp* may play important roles in IB development. Our findings provide some insights into the molecular mechanisms of IB formation, as well as clues for further functional analysis of the identified genes to better understand the development of IBs.

## 1. Introduction

Intermuscular bones (IBs), which only occur in the myosepta of basal teleosts, are small spicule-like bones generated from tendon differentiation [1]. According to the statistics of FAO in 2018, nearly half of the top farmed finfish species worldwide have IBs, such as cyprinids and salmonids [2]. The potential risk of harm to the throat or digestive organs upon consumption greatly reduces the attractiveness of fish with IBs to producers and consumers, and causes obstacles for deep processing, value enhancement, and consumption of such fish [3]. Since the 1960s, IBs have received increasing attention, and extensive research has been carried out on the morphology, ossification process, and number of IBs in different species [3]. Previous studies have also revealed the possibility of reducing the IB number based on ploidy change [4], distant hybridization [5,6], and selective breeding [7]. Notably, an IB-deficient grass carp (*Ctenopharyngodon idella*) was found in an artificial gynogenetic group, and some specimens of tambaqui (*Colossoma macropomum*) without IBs were found in a culture population (normal individuals possess a significant number of IBs) [5,8], indicating the feasibility of genetic improvement of the IB trait. Therefore, the clarification of the molecular mechanisms of IB formation would contribute to the genetic improvement of fish in aquaculture.

Recent advance in high-throughput sequencing technology has greatly facilitated the research on the expression of RNA transcripts in specific tissues or cells, which has significantly improved the understanding of the molecular regulatory mechanisms of the formation of IBs. Recently, comparative transcriptome studies have revealed the molecular characteristics of IB formation and the distinction between IBs and other tissues [9,10]. Additionally, a previous study with histological–transcriptomic–proteomic data suggested that IBs are gradually formed through intramembranous ossification without a cartilaginous phase [11]. Another study compared the orthologous gene families of fish with and without IBs and demonstrated the functional importance of key signaling pathways associated with IB formation [12]. Some studies have characterized the expression of some genes related to IB development, such as *sclerostin* (*sost*) in *Carassius auratus* [13], *tenomodulin* (*tnmd*) and *bone morphogenetic proteins* (*bmps*) in *M. amblycephala* [14], and *muscle segment homeobox 3* (*msx3*) in *Hemibarbus labeo* [15], and the influence of some genes on IB formation has also been verified. For example, scleraxis bHLH transcription factor a (*scxa*) mutation obtained by on the CRISPR/Cas9 system resulted in a clear reduction of mineralized IBs in zebrafish [16], and sp7 transcription factor (*sp7*) mutation in common carp led to shorter IBs [17]. However, many other genes related to bone formation remain uncharacterized during IB development. The formation of IBs may undergo several processes including differentiation of tendon stem/progenitor cells (TSPCs) into osteoblasts, ossification, and mineralization [3,18,19]. Therefore, the genes involved in these processes are worth of exploration to better understand the formation of IBs. It has also been reported that the differentiation of the osteoblast lineage is coordinately regulated by various signaling pathways such as Hedgehog, Notch, Wnt, BMP, and FGF signaling pathways [20,21].

Currently, most studies of IB development, morphology, and transcriptome have been focused on specific fish species with IBs. There is still a lack of comparative studies of myosepta development and transcriptome profiling in fish species with and without IBs. In this study, a comparison analysis was conducted to better understand IB development by using two typical fish species: Blunt snout bream (*Megalobrama amblycephala*), a typical aquaculture species in China belonging to the Cyprinidae family, with a certain number of IBs (epineurals and epipleurals) [11], and Nile tilapia (*Oreochromis niloticus*), an important economic fish belonging to the Perciformes order, without epineurals and epipleurals [22]. We compared the histological structure of the myosepta of these two species from 5 to 60 dph with different staining methods (alcian blue–nuclear red, alizarin red, hematoxylin–eosin (HE), and toluidine blue). Transcriptome sequencing was performed at four key developmental stages of IBs to compare the gene expression between *M. amblycephala* and *O. niloticus*. Then, the expression levels of possible regulatory genes (*scxa*, *scxb*, RUNX family transcription factor 2 (*runx2a*, *runx2b)*, bone gamma-carboxyglutamate protein (*bgp*), *sp7*, collagen type I alpha *2* (*col1a2*), ectonucleoside triphosphate diphosphohydrolase 5 (*entpd5a*, *entpd5b*)*,* phosphate regulating endopeptidase homolog, X-linked (*phex*), alkaline phosphatase (*alpl*), and fibroblast growth factor 23 (*fgf23*)) during IB development were analyzed. Our findings may contribute to a better understanding of IB development in teleosts.

## 2. Materials and Methods

### 2.1. Ethics Statement

All experiments were conducted in accordance with the guidelines of the National Institute of Health Guide for the Care and Use of Laboratory Animals and approved by the Research Ethics Committee, Huazhong Agricultural University, Wuhan, China (HZAUDO-2016-005, 2016-10-26). All efforts were made to minimize fish suffering.

### 2.2. Sample Collection

*M. amblycephala* and *O. niloticus* were obtained from the Fish Breeding Base of the College of Fisheries, Huazhong Agricultural University. The specimens were anesthetized by MS-222 (Sigma, Saint Louis, MO, USA; 100 mg/L) and sterilized with 75% alcohol before tissue collection. After manual removal of the skins, fins, and vertebra, the tail muscles containing IBs were immediately collected under an anatomical lens at eight stages (10, 15, 20, 25, 30, 40, 50, and 60 dph) as described in our previous study [23]. The whole fish larvae at 5 dph were collected as they were too small for the collection of tail muscle tissue, and the dorsal (with epineurals) and abdominal (with epipleurals) muscle tissues were collected from one-year-old *M. amblycephala* and *O. niloticus*. Each group included three samples, and each sample contained muscle tissues from three *M. amblycephala* or *O. niloticus* specimens. All samples were rapidly placed in RNAiso Plus (TaKaRa, Dalian China), refrigerated at 4 °C for 24 h, and stored at –80 °C before total RNA extraction.

### 2.3. Histological Analysis

The caudal peduncle at different developmental stages (5, 10, 15, 20, 25, 30, 40, 50, 60 dph) of *M. amblycephala* and *O. niloticus* was collected and fixed in 4% paraformaldehyde. Then, the post-fixed tissues were decalcified in 0.5 M EDTA until full decalcification (3–7 d) for histological processing. The decalcified tissues were processed by dehydration through a graded series of ethanol (70–100%), cleared in xylene, and then embedded in paraffin blocks. The 5 μm slices were sectioned using a rotary microtome and then stained with HE, alcian blue–nuclear red, alizarin red, and toluidine blue, among which alizarin red was used to detect bone mineralization, alcian blue–nuclear red to visualize the cartilage, and HE and toluidine blue to observe the overall structure of the tissue [24,25,26].

### 2.4. RNA Library Construction and Sequencing

Total RNA was isolated from samples of *M. amblycephala* and *O. niloticus* at 11 stages using RNAiso Plus Reagent (TaKaRa, Dalian, China) according to the manufacturer’s instructions. RNA quality and quantity were measured using 1% agarose gels and NanoDrop 2000 (Thermo Scientific, Waltham, MA, USA), respectively. The RNA samples from four developmental stages of *M. amblycephala* as indicated in our previous published study [23] and the corresponding stages of *O. niloticus* from 15, 25, 30, and 40 dph were used for sequencing analysis. A total of 2 μg of RNA per sample with RIN controlled at 9.6–10 was used as input material for RNA sample preparation. Sequencing libraries were generated using the NEBNext^®^ UltraTM RNA Library Prep Kit for Illumina^®^ (NEB, Ipswich, MA, USA) following the manufacturer’s recommendations, and index codes were added to attribute sequences to each sample. Subsequently, clustering of the index-coded samples was performed on a cBot cluster generation system using the HiSeq PE Cluster Kit v4-cBot-HS (Illumina, San Diego, CA, USA) according to the manufacturer’s instructions. After cluster generation, the libraries were sequenced on an Illumina platform, and 150 bp paired-end reads were generated. Finally, clean data were obtained by filtering the sequencing adapter sequence and low-quality sequencing data.

### 2.5. Differential Expression and Functional Enrichment Analysis

In order to compare the gene expression levels at different developmental stages in *M. amblycephala* and *O. niloticus*, the raw data of four developmental stages of *M. amblycephala* from our previous study [23] were filtered and aligned to the *M. amblycephala* reference genome [9], while the data of *O. niloticus* were aligned to the *O. niloticus* reference genome (https://asia.ensembl.org/Oreochromis_niloticus/Info/Index, accessed on 15 September 2019). The DESeq2 in R packages were used to identify differentially expressed mRNAs with *q*-value < 0.05 and Log2FoldChange > 1. Besides, Gene Ontology (GO) and Kyoto Encyclopedia of Genes and Genomes (KEGG) enrichment analysis of differentially expressed genes (DEGs) were implemented by KOBAS (http://kobas.cbi.pku.edu.cn/kobas3/genelist/, accessed on 24 February 2021).

### 2.6. Gene Expression Analysis

**Figure 1:**
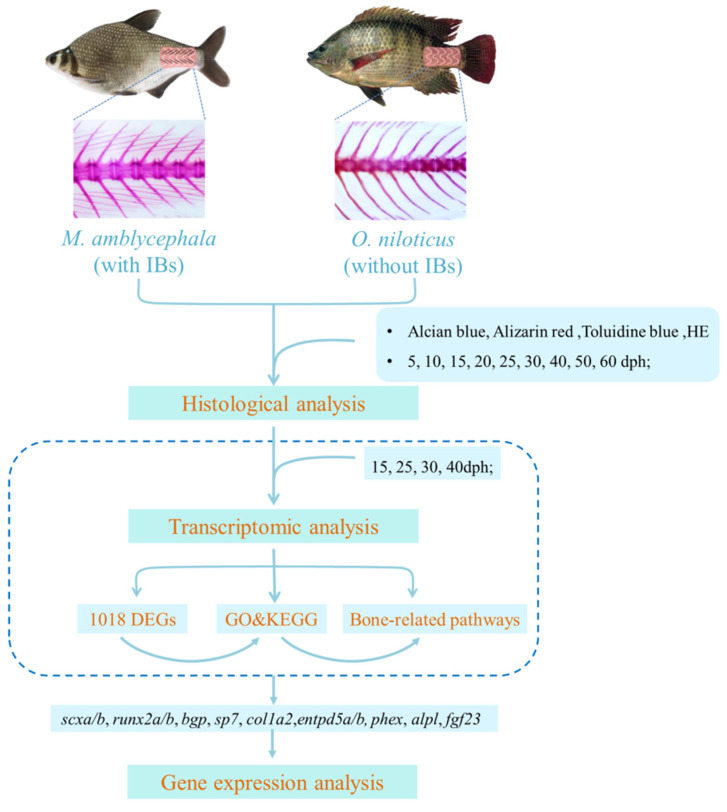
Research scheme diagram.

Reverse-transcription PCR was conducted to synthesize cDNA from 1 μg of total RNA by using the HiScript^®^IIQ RT SuperMix for qPCR (+gDNA wiper) (Vazyme, Nanjing, China) following the manufacturer’s protocol. Quantitative real-time PCR (qRT-PCR) was carried out on a QuantStudio^™^ 6 Flex real-time PCR System (Applied Biosystems, Carlsbad, CA, USA) according to the manufacturer’s instructions. Primer pairs are shown in Table S2. qRT-PCR was performed using SYBR^®^ Premix DimerEraser™ (TaKaRa, Shiga, Japan). The qRT-PCR program consisted of pre-denaturation at 95 °C for 5 min and 40 cycles of amplification at 95 °C for 15 s, 60 °C for 30 s, and 72 °C for 30 s. Each experiment was conducted with three replicates. A housekeeping gene (*β-actin*) was used as a reference gene in quantification analysis. A melting curve analysis was performed at the end of the reaction to demonstrate the specificity of the reaction. For each data analysis, the expression was quantified based on the comparative C_T_ method ($2^{-△△\;C_{T}}$ formula), and the developmental stage or gene corresponding to the largest C_T_ value in the same species was chosen as the reference sample. All values are presented as means ± standard error (SE). One-way ANOVA was conducted using GraphPad Prism8 software to examine the differential expression of genes at different stages in *M. amblycephala* and *O. niloticus*. Statistical significance was accepted at the level of *p* < 0.05. Trend analysis was performed on the hiplot (https://hiplot.com.cn//, accessed on 2 September 2021) using the average value of relative expression level. The entire research scheme diagram is shown in Figure 1.

## 3. Results

### 3.1. Histological Structure

**Figure 2:**
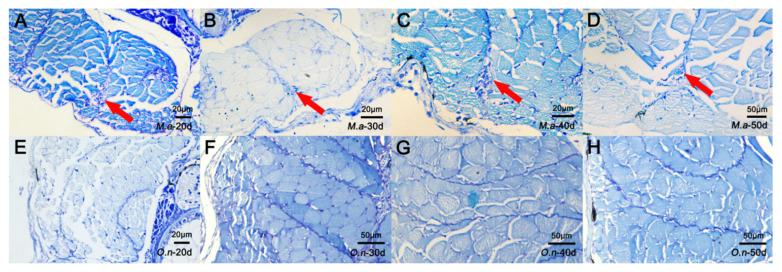
Histological characteristics during IB development based on toluidine blue staining in *M. amblycephala* and *O. niloticus*. (**A**–**D**) Staining at 20, 30, 40, and 50 dph of *M. amblycephala*; (**E**–**H**) staining at 20, 30, 40, and 50 dph of *O. niloticus*. The IBs in *M. amblycephala* are marked by red arrows. No IBs were identified in *O. niloticus*.

**Figure 3:**
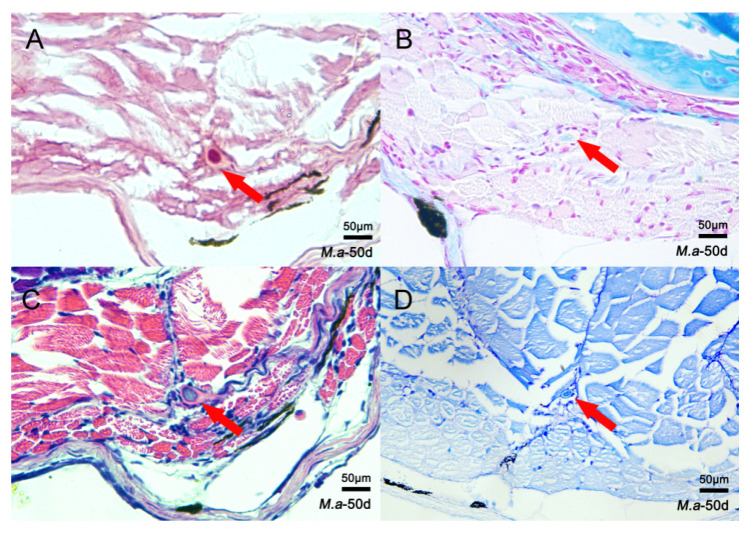
Histological characteristics of *M. amblycephala* IBs based on four staining methods at 50 dph. (**A**) Alizarin red; (**B**) alcian blue–nuclear red; (**C**) HE; (**D**) toluidine blue. The IBs are marked by red arrows.

To identify histological characteristics during IB development, four different staining methods were used to examine changes in tissue structure during the ossification process of IBs. Samples of nine developmental stages were collected from *M. amblycephala* and *O. niloticus*. In *M. amblycephala,* dye condensation was observed in the myosepta 20 dph using toluidine blue staining (Figure 2A). Then, bone-like staining nodules became clearer and larger from 20 to 50 dph with the growth of IBs (Figure 2B–D). At 50 dph, the staining nodules of IBs were clearly visible in the myosepta when performing alizarin red, HE, and toluidine blue staining; however, alcian blue–nuclear red failed to stain the IBs (Figure 3A–D). As for *O. niloticus,* that does not possess IBs, dye condensation and bone-like nodules were not observed in the myosepta at any stage (Figure 2E–H).

### 3.2. Comparative Transcriptome Analysis

**Figure 4:**
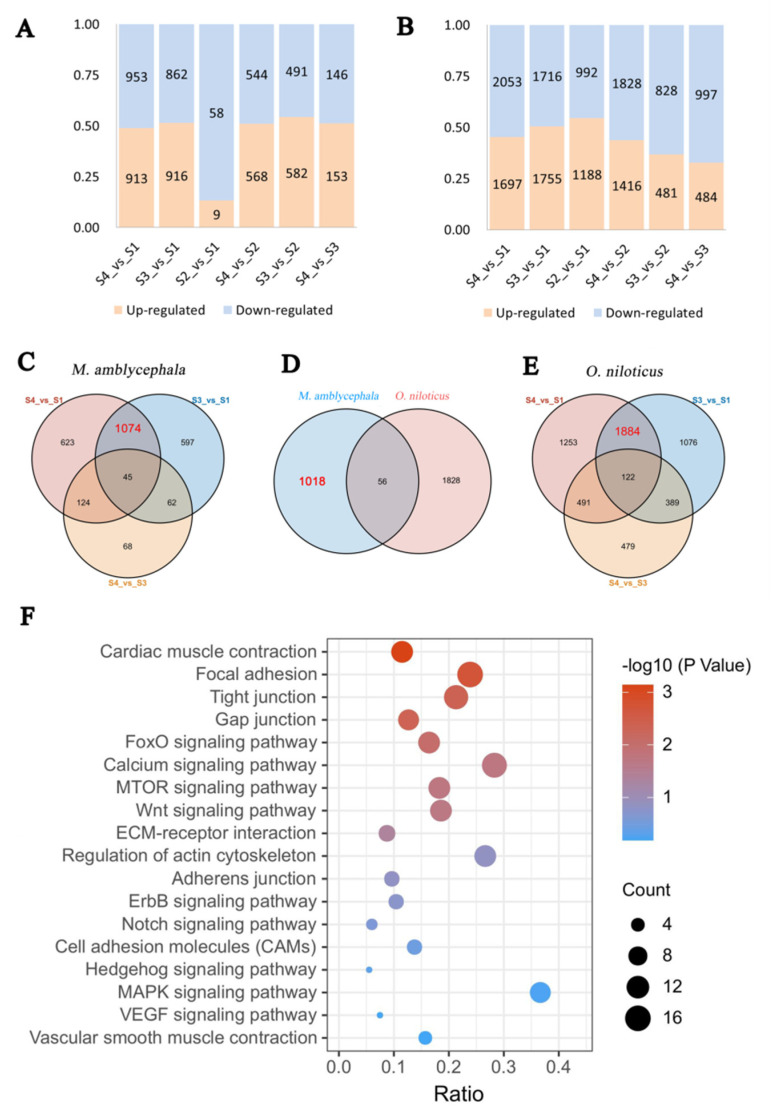
Comparative transcriptome analysis. (**A**) Number of DEGs in *M. amblycephala*; (**B**) number of DEGs in *O. niloticus*; (**C**) Venn diagram for DEGs in three comparison groups in *M. amblycephala*; (**D**) Venn diagram of shared and unique genes between *M. amblycephala* and *O. niloticus*; (**E**) Venn diagram for DEGs in three comparison groups in *O. niloticus*; (**F**) signaling pathways related to bone development enriched in 1018 DEGs identified by comparative transcriptome analysis.

To investigate the genetic regulation of IB development, we compared the transcriptome profiles of *M. amblycephala* and *O. niloticus* at different developmental stages. Figure 4A presents the results from the preliminary analysis of DEGs at different developmental stages of *M. amblycephala*, while Figure 4B presents the analysis results for *O. niloticus*.

In the S2-vs-S1 comparison, 53 DEGs were uniquely found in *M. amblycephala* and not in *O. niloticus* at any stage; these genes may play an important role in the early development of IBs. The Venn diagram revealed that 1074 DEGs were uniquely present in S4-vs-S1 and S3-vs-S1 in *M. amblycephala* (Figure 4C), while 1884 DEGs were uniquely found in S4-vs-S1 and S3-vs-S1 in *O. niloticus* (Figure 4E). Finally, 1018 DEGs present in *M. amblycephala* but not in *O. niloticus* were selected for further analysis (Figure 4D); these genes are very likely associated with the development of IBs. Then, GO enrichment analysis revealed that many of these genes are involved in nucleus (GO:0005634), cytoplasm (GO:0005737), and ATP binding (GO:0005730) (Table S5). The KEGG analysis results demonstrated that these genes were enriched in 133 pathways, including many bone-related pathways, such as focal adhesion, calcium signaling, tight junction, and Wnt signaling pathways (Table S6) (Figure 4F). There were 15, 11, 11, and 10 DEGs in calcium signaling, mTOR signaling, Wnt signaling, MAPK signaling pathways, respectively, which were all related to osteoclast and osteoblast differentiation.

### 3.3. Gene Expression Analysis

**Figure 5:**
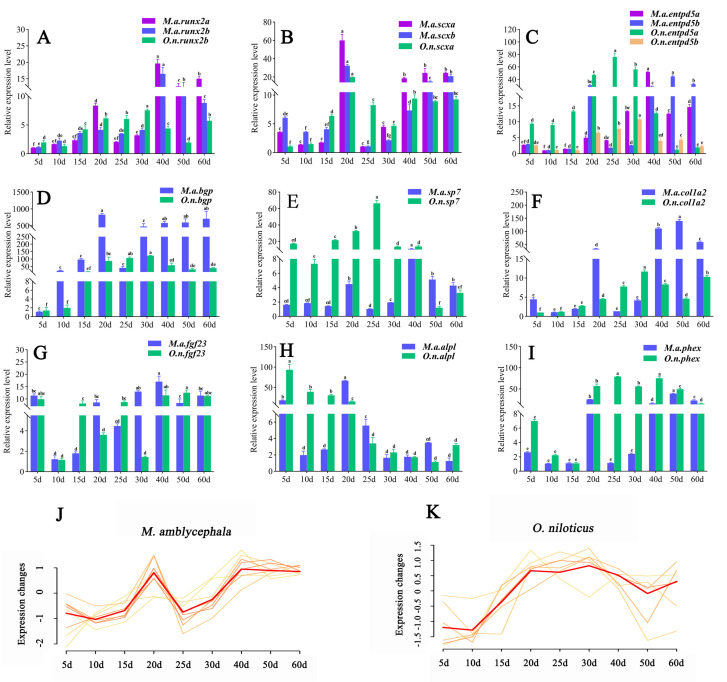
Expression analysis of the tested genes during IB development. (**A**–**I**) Relative expression levels of the tested genes (*runx2*, *scx*, *entpd5, bgp*, *sp7*, *col1a2*, *fgf23*, *alpl*, *phex*) at nine developmental stages in *M. amblycephala* and *O. niloticus*. The same letters above the columns for each gene mean no significant difference (*p* > 0.05); (**J**,**K**) expression trends of *scx*, *runx2*, *entpd5*, *bgp*, and *col1a2* in *M. amblycephala* and *O. niloticus* at nine developmental stages.

The gene expression profiles during the development of *M. amblycephala* and *O. niloticus* were analyzed by qRT-PCR (Figure 5A–K). The relative expression levels of all the tested genes in *M. amblycephala* showed an increasing tendency from 10 dph to 20 dph, particularly those of *scxa*, *scxb*, *bgp*, *col1a2*, and *alpl*. Moreover, from 25 dph to 40 dph, the expression levels of all tested genes still showed an increasing tendency in *M. amblycephala*, except for *alpl*, while they remained unchanged or even declined in *O. niloticus*, suggesting that most of these genes are involved in the formation of IBs. At 40 dph, the relative expression of *runx2a*, *runx2b*, *entpd5a*, *sp7*, and *fgf23* in *M. amblycephala* reached a peak, and the expression of *col1a2* was 103 times that at 10 dph. From 40 dph to 60 dph, the expression of *runx2a*, *runx2b*, *scxa*, *scxb*, *entpd5b*, *bgp*, and *col1a2* was maintained at relatively high levels in *M. amblycephala*, which was not observed in *O. niloticus*. It was thus confirmed that most of the tested genes had higher expression levels in *M. amblycephala* than in *O. niloticus* at the same stage, particularly *scx*, *runx2*, *entpd5*, *col1a2*, and *bgp*. However, *O. niloticus* showed higher expression levels of *alpl*, *sp7*, and *phex* than *M. amblycephala*. There was no significant difference in the expression level of *fgf23* between *M. amblycephala* and *O. niloticus* for most of the tested stages. Taken together, the qRT-PCR analysis revealed that *M. amblycephala* had higher relative expression levels of *scx*, *runx2*, *entpd5*, *col1a2*, and *bgp* at most stages, especially at the late stage of IB development. Interestingly, these genes showed generally the same changing pattern of expression in *M. amblycephala* (Figure 5J), which was completely different from that in *O. niloticus* (Figure 5K).

**Figure 6:**
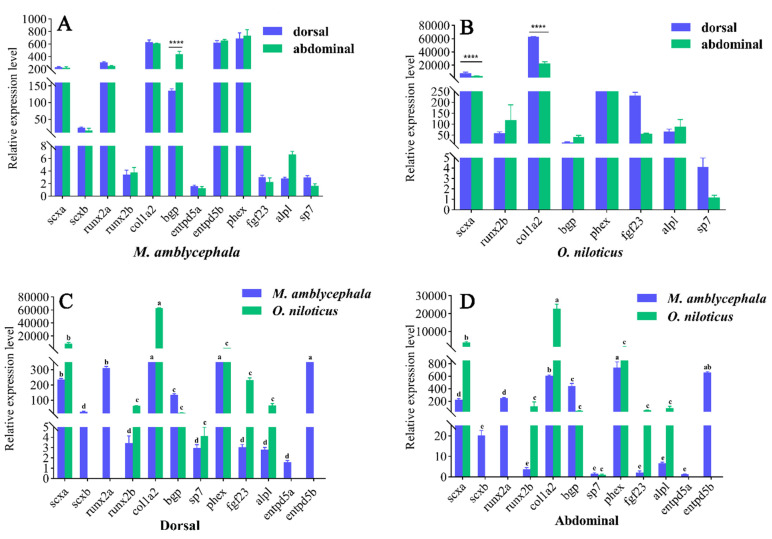
Relative expression levels of the tested genes (*runx2*, *scx*, *entpd5, bgp*, *sp7*, *col1a2*, *fgf23*, *alpl*, *phex*) in the dorsal and abdominal parts in one-year-old *M. amblycephala* and *O. niloticus*. **** indicates an extremely significant difference (*p* < 0.0001); the same letters above the columns for each gene mean no significant difference (*p* > 0.05). (**A**) *M. amblycephala*; (**B**) *O. niloticus*; (**C**) comparison between *M. amblycephala* and *O. niloticus* in the dorsal part; (**D**), comparison between *M. amblycephala* and *O. niloticus* in the abdominal part.

We further characterized the expression of the tested genes in the dorsal and abdominal muscle tissues of *M. amblycephala* and *O. niloticus*. Figure 6A–D shows that there was no significant difference in the expression of most genes between the dorsal and the abdominal part in one-year-old *M. amblycephala* (*p* < 0.05), except for the *bgp* gene, suggesting that *bgp* has a greater impact on epipleurals than on epineurals. In both the dorsal and the abdominal parts, the *scxa, scxb*, *runx2a*, *col1a2*, *bgp*, *entpd5b*, and *phex* genes had relatively higher expression levels than *runx2b*, *entpd5a*, *fgf23*, *alpl*, and *sp7* in *M. amblycephala*. Surprisingly, both *scxa* and *col1a2* showed high expression levels in the dorsal and abdominal parts, and their expression in the dorsal part was significantly higher than that in the abdominal part in one-year old *O. niloticus*. The expression trend of these genes in the dorsal and abdominal parts was basically the same in *M. amblycephala* and *O. niloticus*.

## 4. Discussion

It has been reported that IBs are ossified from myosepta [1]. To better understand the difference in myosepta development between fish with and without IBs, a histological analysis was carried out to compare myosepta development in *M. amblycephala* (with IBs) and *O. niloticus* (without IBs). Toluidine blue staining showed significant differences in myosepta development between *M. amblycephala* and *O. niloticus*, and dye condensation and bone-like nodules were found during myosepta development in *M. amblycephala* but not in *O. niloticus.* The results of alizarin red and alcian blue–nuclear red staining at different developmental stages of *M. amblycephala* further support the conclusion that IBs are membrane bones formed from intramembranous ossification without a cartilaginous phase [11].

In this study, a temporal differential expression analysis was performed to compare gene expression at different developmental stages between *M. amblycephala* and *O. niloticus*, and a total of 53 DEGs were screened. These genes may play important roles in the initial formation of IBs. A preliminary analysis of the transcriptome data in the late period of IB development revealed that 1018 DEGs may be related to IB development. GO and KEGG analyses identified some pathways involved in osteoblast and/or osteoclast development, including calcium, mTOR, MAPK, VEGF, FoxO, and ErbB signaling pathways [27,28,29,30]. A previous review has particularly explored the functions of several molecular signaling pathways during bone formation and development, including Hedgehog signaling, Notch signaling, and Wnt signaling pathways [20]. Three pathways, respectively, for cardiac muscle contraction, regulation of actin cytoskeleton, and vascular smooth muscle contraction have certain correlations with muscle contraction. Previous research has demonstrated that IBs may be associated with the regulation of muscle contraction [12,31,32]. Bone is isolated by a layer of osteoblasts connected by tight and gap junctions with unique cellular functions and complex molecular composition [33]. During the formation of the skeleton structure, junction-associated proteins, which only allow the regulated transport and limit the free diffusion of molecules, are expressed in osteoblasts, generating significant resistance across osteoblast monolayers, while gap junctions may play important roles in the communication between cells through connexins as well as in growth, development, and tissue homeostasis [34,35,36]. Tight junction proteins can transmit signals to the cell interior either directly or by recruiting other signaling molecules to regulate cell proliferation, migration, survival, and differentiation [37]. Cellular interaction requires the support of signaling pathways such as those associated with focal adhesions, ECM–receptor interactions, adherens junctions, tight junctions, Notch, and cell adhesion molecules [38,39,40,41]. Hence, it can be speculated that some of the genes screened in our research may participate in bone formation and development. Although numerous studies have shown that many pathways are involved in skeletal development, no pathway or gene has been found to be specifically essential for IB development, and our study may provide a reference for future research.

As a type of pluripotent stem cells, TSPCs can form tendon-like, bone-like, and tendon-bone junction-like tissues in rat models [42,43]. The development of IBs is a complex biological process, which may involve condensation of pluripotent stem cells, differentiation of osteoblasts, and mineralization [3]. This study analyzed the expression of relevant genes (*scxa*, *scxb*, *runx2a*, *runx2b*, *bgp*, *sp7*, *col1a2*, *entpd5a*, *entpd5b*, *phex*, *alpl*, and *fgf23*) during the development of IBs, with the aim to improve our understanding of the molecular mechanisms of IB development.

*Scx*, a basic helix–loop–helix (bHLH) transcription factor, is relevant to tendon differentiation and maturation as a well-established marker of tendon cells [44,45]. As reported in mammals, the *bgp* gene could promote the differentiation of preosteoblasts into mature osteoblasts [46]. As a Type I collagen, COL1A2 is particularly abundant in tendon and bone tissues [47]. *Alpl*, that encodes alkaline phosphatase (ALP), is highly expressed in the cells of mineralized tissues and plays a critical role in the formation of hard tissues [48]. The expression of these genes showed a dramatic increase at 20 dph in *M. amblycephala*, which is consistent with the histological results showing that dye condensation occurred in the myosepta at 20 dph. These results suggest that these genes may be involved in the early formation of IBs. This finding is consistent with that of a previous study, which reported that the knockout of *scxa* would lead to an obvious reduction of IBs in zebrafish [16].

IBs are mostly composed of osteocytes, bone collagen, and calcium hydroxyapatite [49]. Osteocytes are derived from osteoblasts and then trapped and surrounded by bone matrix. The bone matrix is composed of bone collagen protein synthesized by the osteoblasts and requiring the expression of *col1a2*, non-collagen proteins (such as osteocalcin encoded by *bgp*), which may help to regulate matrix mineralization and affect bone cell activity, and minerals (mainly hydroxyapatite) [50]. The *runx2* gene can promote the differentiation of multipotent mesenchymal cells into osteoblasts [21,51]. Besides, *runx2* induces the expression of *bgp*, encoding osteocalin, to induce the differentiation of preosteoblasts into mature osteoblasts [52,53]. The *entpd5* gene plays an important role in phosphate homeostasis and has been demonstrated to be essential for skeletal mineralization in zebrafish [54,55]. In this study, these genes showed high expression levels from 40 dph to 60 dph (the period of rapid growth and perfection of IBs) in *M. amblycephala*, which may be of great significance to the development of IBs. The *osterix* (*Sp7*) gene can activate the differentiation of preosteoblast cells into mature osteoblast cells and osteocytes [56,57]. However, it is surprising that the expression of *sp7* did not show much change during the formation and development of IBs and, in *M. amblycephala*, was even much lower than in *O. niloticus*.

The PPi/Pi ratio is important for normal bone mineralization. *Alpl* encodes the tissue-non-specific isoenzyme of alkaline phosphatase (TNSALP), which dephosphorylates PPi into inorganic Pi [58]. The *phex* gene can increase the concentration of Pi in the ECM by promoting renal Pi reabsorption, while *fgf23* has an opposite effect [59,60]. There was no significant change in the expression levels of these genes during 40–60 dph in *M. amblycephala* and *O. niloticus*, but the expression of these genes in the dorsal and abdominal muscles of one-year-old *O. niloticus* was higher than in the corresponding parts of one-year-old *M. amblycephala*. However, in the dorsal and abdominal parts of one-year-old *M. amblycephala,* the *phex*/*fgf23* expression ratios were 229 and 324, respectively, much higher than in *O. niloticus* (6 and 22, respectively). The higher relative expression of *phex* with respect to *fgf23* is conducive to Pi deposition and may promote the production of hydroxyapatite crystals during IB development. These genes may have important effects on the formation of IBs, which needs to be further validated considering their higher expression in *O. niloticus*.

In general, our quantitative results showed that *scx*, *runx2*, *entpd5*, *col1a2*, and *bgp* have higher relative expression levels at most developmental stages of *M. amblycephala*, particularly at the late stage of IB development.

## 5. Conclusions

IBs are characterized as spicule-like bones existing in basal teleosts, with uncertain functions. In this study, histological staining was combined with RNA-Seq to reveal differences in myosepta development between *M. amblycephala* and *O. niloticus*. First, histological changes in IBs during the development of *M. amblycephala* were revealed, and it was found that IB development does not involve the cartilage phase. Then, the dynamics of molecular features in developing IBs were analyzed, and a number of candidate genes were screened according to the literature. Finally, the functions of these candidate genes in IB development were characterized. Our results suggest that the *scxa*, *scxb*, *runx2a*, *runx2b*, *entpd5a*, *col1a2*, and *bgp* genes may play important roles in the formation, ossification, and mineralization of IBs. Overall, our findings contribute to a better understanding of IB formation and development and provide an important reference for further understanding the molecular mechanisms of IB formation. In future research, gene knockout or RNA interference may be employed to elucidate gene functions during IB development in fish.

## Data Availability

All transcriptome data of tilapia are available in the NCBI database under Accession number PRJNA729911. All transcriptome data of blunt snout bream are available in the NCBI database under Accession number PRJNA263980.

## Supplementary Materials

The following are available online at https://www.mdpi.com/article/10.3390/biology10121311/s1, Table S1: Amino acid sequence GeneBank accession number/Ensembl ID of bone-related genes in different species, Table S2: Primer sequences for genes qRT-PCR of *M. amblycephala* and *O. niloticus*, Table S3: List of DEGs in different comparison groups of *M. amblycephala*, Table S4: List of DEGs in different comparison groups of *O. niloticus*, Table S5: GO terms of 1018 DEGs uniquely shared in S4_vs_S1 and S3_vs_S1 in *M. amblycephala*, Table S6: KEGG pathway information of 1018 DEGs uniquely shared in S4_vs_S1 and S3_vs_S1 in *M. amblycephala*.
